# The PapB/FocB family protein TosR acts as a positive regulator of flagellar expression and is required for optimal virulence of uropathogenic *Escherichia coli*

**DOI:** 10.3389/fmicb.2023.1185804

**Published:** 2023-07-18

**Authors:** Hidetada Hirakawa, Mizuki Shimokawa, Koshi Noguchi, Minori Tago, Hiroshi Matsuda, Ayako Takita, Kazutomo Suzue, Hirotaka Tajima, Ikuro Kawagishi, Haruyoshi Tomita

**Affiliations:** ^1^Department of Bacteriology, Graduate School of Medicine, Gunma University, Maebashi, Gunma, Japan; ^2^Department of Infectious Diseases and Host Defense, Graduate School of Medicine, Gunma University, Maebashi, Gunma, Japan; ^3^Department of Frontier Bioscience and Research Center for Micro-Nano Technology, Hosei University, Tokyo, Japan; ^4^Laboratory of Bacterial Drug Resistance, Graduate School of Medicine, Gunma University, Maebashi, Gunma, Japan

**Keywords:** urinary tract infection, bacterial pathogenesis, virulence, flagella, fimbriae, Biofilm

## Abstract

Uropathogenic *Escherichia coli* (UPEC) is a major causative agent of urinary tract infections. The bacteria internalize into the uroepithelial cells, where aggregate and form microcolonies. UPEC fimbriae and flagella are important for the formation of microcolonies in uroepithelial cells. PapB/FocB family proteins are small DNA-binding transcriptional regulators consisting of approximately 100 amino acids that have been reported to regulate the expression of various fimbriae, including P, F1C, and type 1 fimbriae, and adhesins. In this study, we show that TosR, a member of the PapB/FocB family is the activator of flagellar expression. The *tosR* mutant had similar expression levels of type 1, P and F1C fimbriae as the parent strain, but flagellar production was markedly lower than in the parent strain. Flagellin is a major component of flagella. The gene encoding flagellin, *fliC*, is transcriptionally activated by the sigma factor FliA. The *fliA* expression is induced by the flagellar master regulator FlhDC. The *flhD* and *flhC* genes form an operon. The promoter activity of *fliC, fliA* and *flhD* in the *tosR* mutant was significantly lower than in the parent strain. The purified recombinant TosR does not bind to *fliC* and *fliA* but to the upstream region of the *flhD* gene. TosR is known to bind to an AT-rich DNA sequence consisting of 29 nucleotides. The characteristic AT-rich sequence exists 550–578 bases upstream of the *flhD* gene. The DNA fragment lacking this sequence did not bind TosR. Furthermore, loss of the *tosR* gene reduced motility and the aggregation ability of UPEC in urothelial cells. These results indicate that TosR is a transcriptional activator that increases expression of the *flhDC* operon genes, contributing to flagellar expression and optimal virulence.

## Introduction

Urinary tract infection (UTI) is one of the most common infectious diseases. Uropathogenic *Escherichia coli* (UPEC) is the most major causative agent of UTIs in the community, with more than 80% of cases caused by this organism (Zhang and Foxman, 2003; Flores-Mireles et al., 2015). Antimicrobial agents are commonly used to treat infections, however drug-resistant UPEC such as quinolone-resistant and extended-spectrum β-lactamase (ESBL)-producing strains have increased in recent years (Mathers et al., 2015; Walker et al., 2016). For these reasons, the development of treatments for UPEC infections is desirable.

UPEC invades into the urinary tract cells, where they aggregate and form biofilm-like microbial colonies termed intracellular bacterial community (IBC; Anderson et al., 2003). This ability allows UPEC to escape various antimicrobial agents and the immune system (Mulvey et al., 1998, 2001). Hence, it is believed that UPEC infections are more likely to become refractory and recurrent.

Fimbriae and flagella are major protein structures contributing to the virulence of UPEC. Fimbriae, such as type 1, P and F1C fimbriae, are required for bacterial adhesion to and invasion of host urothelial cells (Virkola et al., 1988; Martinez et al., 2000; Lillington et al., 2014). Flagella are required for bacterial migration to infection sites as the bacteria colonize the bladder and kidneys, and flagellum-mediated motility contributes to bacterial fitness and aggregation in infected urinary tract cells (Lane et al., 2005; Wright et al., 2005; Hirakawa et al., 2019). In a study using a UTI mouse model, UPEC flagella were shown to be highly expressed within infected mice (Lane et al., 2007). Another studies showed that the flagellum-deficient strain had lower ability to invade bladder and kidney epithelial cells and to form microcolonies compared to the flagellum-producing strain (Pichon et al., 2009; Hirakawa et al., 2019). In addition to fimbriae and flagella, hemolysin is another important UPEC virulence factor. This protein, a type of pore-forming toxin, contributes to the development of cystitis, pyelonephritis, and sepsis by destroying immune system cells such as macrophages in addition to urinary tract epithelial cells (Dhakal and Mulvey, 2012).

We are interested in characterizing proteins involved in the pathogenicity of UPEC including bacterial aggregation leading to IBC formation, thereby contributing to the elucidation of the pathogenesis of this organism and the development of novel therapeutic strategies. PapB and FocB are DNA-binding transcriptional regulators of small size consisting of approximately 100 amino acids. They have been reported to regulate the expression of P and F1C fimbriae (Forsman et al., 1989; Lindberg et al., 2008). We studied the *tosR* gene found on the pathogenicity island (PAI) of the UPEC CFT073 strain. This strain was isolated from the blood and urine of a patient with acute pyelonephritis. The *tosR* gene encodes the PapB/FocB family transcriptional regulator and is part of an operon that includes the *tosC*, *tosB*, and *tosD* genes downstream (Welch et al., 2002). The *tosC*, *tosB*, and *tosD* genes were originally presumed to encode a group of proteins of the type 1 secretion system that contribute to hemolysin secretion. The *tosA* gene, encoding a repeat-in-toxin (RTX) nonfimbrial adhesin, is present downstream of the *tosRCBD* operon, and TosC, TosB and TosD were shown to contribute to the secretion of TosA (Lloyd et al., 2009). TosR has been shown to contribute to the formation of Auf fimbriae and biofilm (Vigil et al., 2012; Luterbach et al., 2018). However, the function of TosR remains understudied.

This study demonstrated that the TosR protein/regulator contributes to UPEC’s ability to aggregate in bladder epithelial cells. The gene deletion mutant had lower motility and flagellar expression and exhibited lower levels of aggregation within the bladder epithelial cells. However, P and F1C fimbriae and hemolysin activity were at the same levels as in the parent strain. The purified TosR protein bound to upstream region of *flhD* which encodes the master regulator for flagellar expression. Thus, TosR is an activator for UPEC virulence-associated flagellar expression.

## Materials and methods

### Bacterial strains and culture conditions

The bacterial strains and plasmids used in this study are listed in Table 1, 2, respectively. All bacteria were grown in Luria-Bertani (LB) medium. The cell growth was monitored by absorbance at 600 nm. For marker selection and maintenance of plasmids, antibiotics were added to growth media at the following concentrations; 150 μg/ml ampicillin, 30 μg/ml chloramphenicol, and 50 μg/ml kanamycin.

**Table 1:** Bacterial strains used in this study.

Strains	Relevant genotype/phenotype	Reference
CFT073	Parent strain (ATCC 700928)	ATCC 700928
CFT073∆tosR	*tosR* mutant from CFT073	This work
CFT073∆tosRCBD	*tosRCBD* mutant from CFT073	This work
CFT073∆tosCBD	*tosCBD* mutant from CFT073	This work
Rosetta™(DE3)	T7-expression strain, Cm^R^	Novagen

Cm^R^, Chloramphenicol resistance.

**Table 2:** Plasmids used in this study.

Plasmids	Relevant genotype/phenotype	Reference
pKO3	Temperature sensitive vector for gene targeting, *sacB*, Cm^R^	Link et al. (1997)
pTrc99A	Vector for IPTG-inducible expression; Ap^R^	Hirakawa et al. (2003a)
pTrc99AtosR	*tosR* expression plasmid; Ap^R^	This work
pTrc99K	Vector for IPTG-inducible expression; Km^R^	Hirakawa et al. (2003b)
pTrc99KtosR	*tosR* expression plasmid; Km^R^	This work
pTurboGFP-B	GFP expression plasmid; Ap^R^	Evrogen
pNN387	Single-copy plasmid with promoterless *lacZ*, Cm^R^	Elledge and Davis (1989)
pNNflhD-P-Long	*flhD* promoter reporter (The region from 769-bp upstream from the *flhD* start codon); Cm^R^	This work
pNNflhD-P-Short	*flhD* promoter reporter (The region from 300-bp upstream from the *flhD* start codon); Cm^R^	This work
pNNfliA-P	*fliA* promoter reporter; Cm^R^	Hirakawa et al. (2021)
pNNfliC-P	*fliC* promoter reporter; Cm^R^	Hirakawa et al. (2021)
pTH18kr	Low copy plasmid; Km^R^	Hashimoto-Gotoh et al. (2000)
pTH18krfliC-VSVG	C-terminally VSVG-tagged FliC expression plasmid; Km^R^	Hirakawa et al. (2021)
pTH18krtosR	*tosR* expression plasmid; Km^R^	This study
pET42c	Vector for expression of His-tagged proteins; Km^R^	Novagen
pET42ctosR	C-terminal TosR-His_8_ overexpression plasmid; Km^R^	This study

Cm^R^, Chloramphenicol resistance, Ap^R^, Ampicillin resistance, Km^R^, Kanamycin resistance.

### Cloning and mutant constructions

An in-frame deletion mutant of *tosR* (CFT073∆tosR) was constructed by sequence overlap extension PCR according to a strategy described previously (Link et al., 1997), with primer pairs, delta1 / delta2 and delta3 /delta4 primers as described in Table 3. The upstream flanking DNA included 450 bp and the first eight amino acid codons. The downstream flanking DNA included the last one amino acid codon, the stop codon, and 450 bp of DNA. This deletion construct was ligated into BamHI and SalI-digested temperature sensitive vector pKO3 (Link et al., 1997) and introduced into the chromosome of CFT073 strain. Then, sucrose-resistant/chloramphenicol-sensitive colonies were selected at 30°C. To construct CFT073∆tosRCBD and CFT073∆tosCBD, the locus of *tosR* to *tosD* and *tosC* to *tosD* was deleted using primers, tosR-delta1/tosRD-delta2/tosRD-delta3/tosD-delta4 and tosC-delta1/tosCD-delta2/tosCD-delta3/tosD-delta4, respectively. To construct isopropyl β-D-thiogalactopyranoside (IPTG)-inducible *tosR* expression plasmids, pTrc99AtosR and pTrc99KtosR, the *tosR* gene was amplified with the primer pairs shown in Table 3. The product was digested with NcoI and SalI and ligated into similarly digested pTrc99A and pTrc99K plasmid (Hirakawa et al., 2003a,b), respectively. The pTH18krtosR plasmid was constructed by amplifying *tosR* and the 300-bp upstream region including the promoter and ligating the product into pTH18kr (Hashimoto-Gotoh et al., 2000). Since this plasmid is compatible with pTurboGFP-B, these plasmids were introduced together into the *tosR* mutant to characterize bacterial colonies within bladder epithelial cells using fluorescent images. To construct pNNflhD-P-Long and pNNflhD-P-Short, lacZ reporter plasmids, 769-bp and 300-bp upstream region of the *flhD* gene were amplified using primer pairs flhD-PFLong/flhD-PREcoRV and flhD-PFShort/flhD-PRHindIII and cloned into the pNN387 plasmid with a promoterless *lacZ* (Elledge and Davis, 1989), respectively. We also constructed an IPTG-inducible C-terminal octahistidine-tagged TosR expression plasmid, pET42ctosR. The *tosR* gene was amplified using pETtosR-F and pETtosR-R primers, and ligated into the pET42c vector digested with NdeI and HindIII. All constructs were confirmed by DNA sequencing.

**Table 3:** Primers used in this study.

Primers	DNA sequence (5′–3′)	Use
tosR-delta1	gcgggatccgattaactacaaccaatctgg	*tosR* deletion
tosR-delta2	ttgtccgtgcttttgcactaaaaatgatctgccataccattac	*tosR* deletion
tosR-delta3	tgtgtaatggtatggcagatcatttttagtgcaaaagcacggac	*tosR* deletion
tosR-delta4	gcggtcgactgagtgcatcaagatataatg	*tosR* deletion
tosRD-delta2	aatcatcgttcctgaagagcttgatgatctgccataccattac	*tosRCBD* deletion
tosRD-delta3	tgtgtaatggtatggcagatcatcaagctcttcaggaacgatg	*tosRCBD* deletion
tosC-delta1	gcgggatccattgaataatgtgtaatgg	*tosCBD* deletion
tosCD-delta2	aatcatcgttcctgaagagcttgatcgtgcataactgcctccc	*tosCBD* deletion
tosCD-delta3	ttagggaggcagttatgcacgatcaagctcttcaggaacgatg	*tosCBD* deletion
tosD-delta4	gcggtcgacgccgtacactgtgtttcccc	*tosCBD* and *tosRCBD* deletion
pTrc-tosR-F	gcgccatgggtaatggtatggcagatc	pTrc99AtosR and pTrc99KtosR constructions
pTrc-tosR-R	gcggtcgacgtgattgtccgtgcttttgc	pTrc99AtosR and pTrc99KtosR constructions
pTH-tosR-F	gcgggatccgtcgctacatgatggataac	pTH18krtosR construction
pTH-tosR-R	gcgaagcttgtgattgtccgtgcttttgc	pTH18krtosR construction
pET-tosR-F	aagctcatatgtgtaatggtatggcag	pET42ctosR construction
pET-tosR-R	gcgaagcttaaaactattattataatattcac	pET42ctosR construction
flhD-PFLong	gcggcggccgcaacctgttccttattctgtg	pNNflhD-P-Long construction/gel shift assay
flhD-PREcoRV	aagcggatatctattcccacccagaataacc	pNNflhD-P-Long construction/gel shift assay
flhD-PFShort	gcggcggccgcatttatgttaagtaattgagcg	pNNflhD-P-Short construction/gel shift assay
flhD-PRHindIII	gcgaagctttattcccacccagaataacc	pNNflhD-P-Short construction/gel shift assay
fliA-PF	gcggcggccgcctgagactgacggcaacgcc	Gel shift assay
fliA-PR	gcgaagcttcacgataaacagccctgcg	Gel shift assay
fliC-PF	gcggcggccgccctgacccgactcccagcg	Gel shift assay
fliC-PR	gcgaagcttgattcgttatcctatattgc	Gel shift assay
rrsA-qPCR-F	cggtggagcatgtggtttaa	Quantitative PCR
rrsA-qPCR-R	gaaaacttccgtggatgtcaaga	Quantitative PCR
rpoD-qPCR-F	caagccgtggtcggaaaa	Quantitative PCR
rpoD-qPCR-R	gggcgcgatgcacttct	Quantitative PCR
fliC-qPCR-F	tccactgaaagctctggatgaa	Quantitative PCR
fliC-qPCR-R	cccagggatgaacggaatt	Quantitative PCR
fliA-qPCR-F	cgagcgtggaacttgacgat	Quantitative PCR
fliA-qPCR-R	cgacggcattaagtaacccaat	Quantitative PCR
flhD-qPCR-F	gacaacgttagcggcactga	Quantitative PCR
flhD-qPCR-R	ttgattggtttctgccagctt	Quantitative PCR
fimA-qPCR-F	tgcgggtagcgcaacaa	Quantitative PCR
fimA-qPCR-R	acgcagtccctgttttatcca	Quantitative PCR
papA-qPCR-F	tttttcgggtgtcccaagtg	Quantitative PCR
papA-qPCR-R	tgttgcaccgacggtctgt	Quantitative PCR
papA2-qPCR-F	gattgttgttactgattcgagtggaa	Quantitative PCR
papA2-qPCR-R	ccggtctcagtggctccat	Quantitative PCR
focA-qPCR-F	caggcggtttactacgcaact	Quantitative PCR
focA-qPCR-R	cgtcggcgttggcaata	Quantitative PCR
hlyA-qPCR-F	ggcacggcgattactaaacag	Quantitative PCR
hlyA-qPCR-R	cgttcggtgaggccaatg	Quantitative PCR
flgB-qPCR-F	tcaggctcgcgatatcgatt	Quantitative PCR
flgB-qPCR-R	ccgtccacgttgcatgact	Quantitative PCR
flhB-qPCR-F	tcgcgctgcgcattc	Quantitative PCR
flhB-qPCR-R	ttcaagcgtcgggacgtta	Quantitative PCR
fliL-qPCR-F	actggcattcgcatcaggtt	Quantitative PCR
fliL-qPCR-R	ggcacgacgcgttgct	Quantitative PCR
motA-qPCR-F	tgcagggattgggtcgtt	Quantitative PCR
motA-qPCR-R	cgtgcctttaatcgctttgc	Quantitative PCR

### Biofilm assays using 96-well plates

Levels of biofilm formation on 96-well plates were quantified as described previously with slight modifications (Hirakawa et al., 2019). Bacteria were cultured at 30°C for 24 h in LB medium without shaking. Each culture was diluted into LB medium containing 0.5% glucose at a 1:100 ratio, and 2.4 × 10^4^ cells/well were seeded into the VIOLAMO 96-well flat bottom polystyrene plate (As ONE Corp., Osaka Japan, Catalogue number: 1–1,601-02). The plate was then incubated at 30°C for 24 h. Bacterial cells attached to the plate were stained with crystal violet and absorbance at 595 nm (A_595_) was measured. Bacterial aggregation ability was quantified as the A_595_ normalized to an OD_600_ of 1.

### Infection of bladder epithelial cells

The ability of UPEC to invade bladder epithelial cells (HTB-9) was assessed by gentamicin protection assay as previously described (Hirakawa et al., 2019). We inoculated ~5.0 × 10^6^ bacteria into ~5.0 × 10^5^ host cells. The numbers of invaded bacterial cells were determined as ratios of CFU (as percentages) to total cell (invaded and non-invaded bacterial cells) CFU. We also imaged the bacteria in HTB-9 cells using confocal microscopy, as previously described (Hirakawa et al., 2019). A UPEC strain carrying a green fluorescence protein (GFP) expression plasmid, pTurboGFP-B (Evrogen, Moscow, Russia), was inoculated into cultured HTB-9 cells. The HTB-9 cells were stained with rhodamine-phalloidin (Life Technologies, Carlsbad, CA, USA). Fluorescent images were acquired on an Olympus FV10i-DOC microscope and processed using FV10-ASW software (Olympus Corp., Tokyo, Japan).

### Hemagglutination assays

To estimate the activity of type 1 and P fimbriae, we tested the hemagglutination titers of guinea pig and human O-type erythrocytes, respectively, as previously described (Hirakawa et al., 2019).

### Motility assays

Bacteria were statically grown overnight at 37°C. The bacterial cultures (2 μl) were spotted onto LB medium containing 0.3% agar and incubated for 9 h at 30°C.

### Flagellum staining

Bacteria were cultured for 24 h at 30°C in Heart Infusion medium containing 1.5% agar. Flagella were stained with Victoria blue/tannic acid solution as previously described (Hirakawa et al., 2019).

### RNA extraction and quantitative real-time PCR analyses

Bacteria were grown to the late-logarithmic growth phase (OD_600_ ~ 0.7) with shaking in LB medium. Total RNA extraction, cDNA synthesis and real-time PCR were performed as previously described (Hirakawa et al., 2021). Constitutively expressed *rrsA* and *rpoD* genes were used as an internal control. Primers are listed in Table 3.

### Western blotting

To detect VSVG-tagged FliC from UPEC, bacteria were grown to early stationary phase and separated by centrifugation. The cell pellets were resuspended in 50 mM phosphate buffer containing 8 M urea and then lysed by sonication. Cell lysate (4.0 μg) proteins were separated on duplicate 10% acrylamide Tris-glycine SDS-PAGE gels. One gel was stained with Coomassie brilliant blue (CBB), and the other was electroblotted onto a polyvinylidene fluoride (PVDF) membrane (Bio-Rad Laboratories, Hercules, CA). VSVG-tagged FliC was detected with the Sigma anti-VSVG antibody (Merck KGaA, Darmstadt, Germany, Catalogue number: V4888) and the Sigma anti-rabbit horseradish peroxidase-conjugated immunoglobulin G (IgG) secondary antibody (Merck KGaA, Darmstadt, Germany, Catalogue number: A6154) using a SuperSignal West Pico kit (Thermo Fisher Scientific, Waltham, MA). VSVG-tagged FliC protein bands were visualized using a LAS-4010 luminescent image analyzer (GE Healthcare Japan, Tokyo).

### Promoter assay

To measure promoter activities of *flhD*, *fliA* and *fliC*, UPEC strains carrying pNNflhD-P-Long, pNNflhD-P-Short, pNNfliA-P, or pNNfliC-P were grown at 37°C in LB medium. β-Galactosidase activities from *lacZ* expression in cell lysates were determined using a Tropix Galacto-Light Plus kit (Thermo Fisher Scientific, Waltham, MA, United States) as previously described (Hirakawa et al., 2018). The β-galactosidase activity was determined as the signal value normalized to an OD_600_ of 1.

### Purification of TosR

C-terminally octahistidine-tagged TosR (TosR-His_8_) was purified from *Escherichia coli* Rosetta (DE3; Novagen, Philadelphia, PA). Bacteria containing the pET42ctosR plasmid were cultured at 37°C to logarithmic phase in LB medium, 0.5 mM IPTG was then added, and culture growth was continued at 16°C for 18 h. The cells were lysed using the BactYeastLysis reagent (ATTO, Tokyo, Japan) as previously described (Kurabayashi et al., 2016). An equal volume of purification buffer (50 mM Tris [pH 7.5], 200 mM NaCl, and 5% glycerol) was added to the lysate, and the mixture was centrifuged. The resulting supernatant was mixed with nickel-nitrilotriacetic acid (Ni-NTA) agarose (Qiagen, Valencia, CA) for 1 h. The agarose was washed three times with 10 mM imidazole and twice with 50 mM imidazole, and TosR-His_8_ was then eluted with 500 mM imidazole. The protein concentration was determined using a Bio-Rad protein assay (Bio-Rad, Hercules, CA).

### Gel-shift assay

We used a 791-bp DNA probe containing the 769-bp region upstream of *flhD*. The DNA probe was prepared by PCR-amplifying with flhD-PFLong and flhD-PREcoRV primers, listed in Table 4. To generate the DNA probe lacking the TosR binding motif present upstream of *flhD*, flhD-PFShort and flhD-PRHindIII primers were used for PCR-amplification. This probe contains only the 300-bp region upstream of *flhD*. We also prepared for 321-bp DNA probes containing the 300-bp regions upstream of the *fliA* and *fliC* start codons by PCR-amplifying with primer pairs fliA-PF/fliA-PR and fliC-PF/fliC-PR, respectively. The probe fragments (0.30 pmol) were mixed with purified TosR-His_8_. After incubation for 20 min at room temperature, samples were separated by electrophoresis on a 5% nondenaturing acrylamide Tris-borate/EDTA (89 mM Tris-borate [pH 8.3], and 2 mM EDTA) gel in Tris-borate/EDTA buffer at 4°C. DNA bands in the gel were stained by 20,000-fold-diluted Novel Green Plus (BIO-HELIX Co., LTD., Keelung City Taiwan), and visualized under UV light at 300 nm.

**Table 4:** HA titers of the wild-type parent and the tosR mutant.

Strains	HA titers of guinea pig erythrocytes	
	−Mannose	+Mannose
Wild-type (CFT073)	32	<2
*tosR* mutant CFT073ΔtosR	32	<2
Strains	HA titers of human erythrocytes	
	−Mannose	+Mannose
Wild-type (CFT073)	64	64
*tosR* mutant CFT073ΔtosR	64	64

### Statistical analysis

The *p* value in each assay was determined by the unpaired t test with GraphPad Prism version 6.00.

## Results

### The *tosR* gene contributes to UPEC aggregation within uroepithelial cells

**Figure 1:**
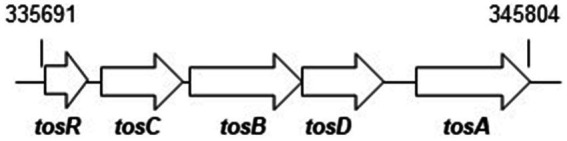
Locus of *tosRCBDA* on the UPEC CFT073 chromosome. Arrows indicate transcription/translation direction. Numbers refer to nucleotide coordinates in the UPEC CFT073 genome.

**Figure 2:**
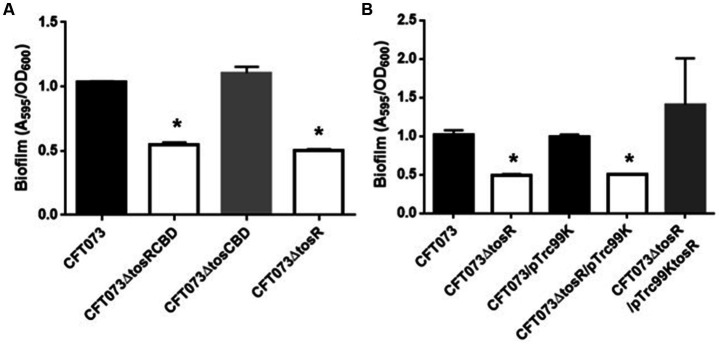
Biofilm formation on 96-well plates in the parent strain (CFT073) and its derivatives **(A)**, or the parent and the tosR mutant carrying pTrc99K (empty vector) or pTrc99KtosR (*tosR* expression plasmid; **B**). Bacterial adhesion and aggregation were represented as A_595_ values normalized to OD_600_ of 1. Data plotted are the means of three biological replicates; error bars indicate standard deviations. *, *P*,0.01 relative to values for CFT073 **(A)** or CFT073/pTrc99K **(B)**.

To investigate the role of TosR, we constructed a CFT073∆tosR strain lacking *tosR*. The *tosR* gene is speculated to form an operon together with the downstream *tosC*, *tosB* and *tosD* genes. TosC, TosB, and TosD were reported to be involved in the secretion of the adhesin TosA (Figure 1; Vigil et al., 2012). To clarify whether TosR, together with TosC, TosB, and TosD, is involved in the adhesion capacity of UPEC, we also constructed CFT073∆tosCBD strain with deletion from the start codon of *tosC* to the stop codon of *tosD* and CFT073∆tosRCBD strain with deletion from the start codon of *tosR* to the stop codon of *tosD*. As part of the evaluation of the adhesion capacity of UPEC, a static biofilm assay was performed. TosR overproduction has been reported to promote biofilm formation, however the *tosR* mutant formed biofilms comparable to the wild-type strain (static cultures at 37°C; Luterbach et al., 2018). In our experimental conditions (Bacteria were cultured at 30°C because our CFT073 strain poorly produced biofilms when cultured at 37°C), the *tosR* mutant (CFT073∆tosR) had a lower biofilm forming ability than the parent strain (CFT073). When the CFT073 parent strain, CFT073∆tosR, CFT073∆tosCBD, and CFT073∆tosRCBD were cultured on 96-well polystyrene plates for 24 h, the CFT073∆tosRCBD and CFT073∆tosR strains, but not CFT073∆tosCBD, showed approximately half the biofilm forming ability of the parent strain (Figure 2A). To confirm the contribution of the *tosR* gene to biofilm formation, we complemented the *tosR* gene to the CFT073∆tosR strain by introducing the pTrc99KtosR plasmid and examined its ability to form biofilms. The pTrc99KtosR plasmid allows *tosR* expression in an IPTG-inducible promoter-dependent manner. Initially, we performed the complementation experiment with 0.01 mM and 0.1 mM of IPTG. Even at a concentration of 0.01 mM IPTG, the complementation of biofilm formation capacity by the addition was observed (Figure 2B). Then, we observed that the bacterial growth in the 0.1 mM culture was noticeably lower, as indicated by the OD_600_ values, compared to both the non-complementary strain and the 0.01 mM culture. Due to this finding, subsequent experiments involving the pTrc99KtosR plasmid were conducted with the addition of 0.01 mM IPTG.

**Figure 3:**
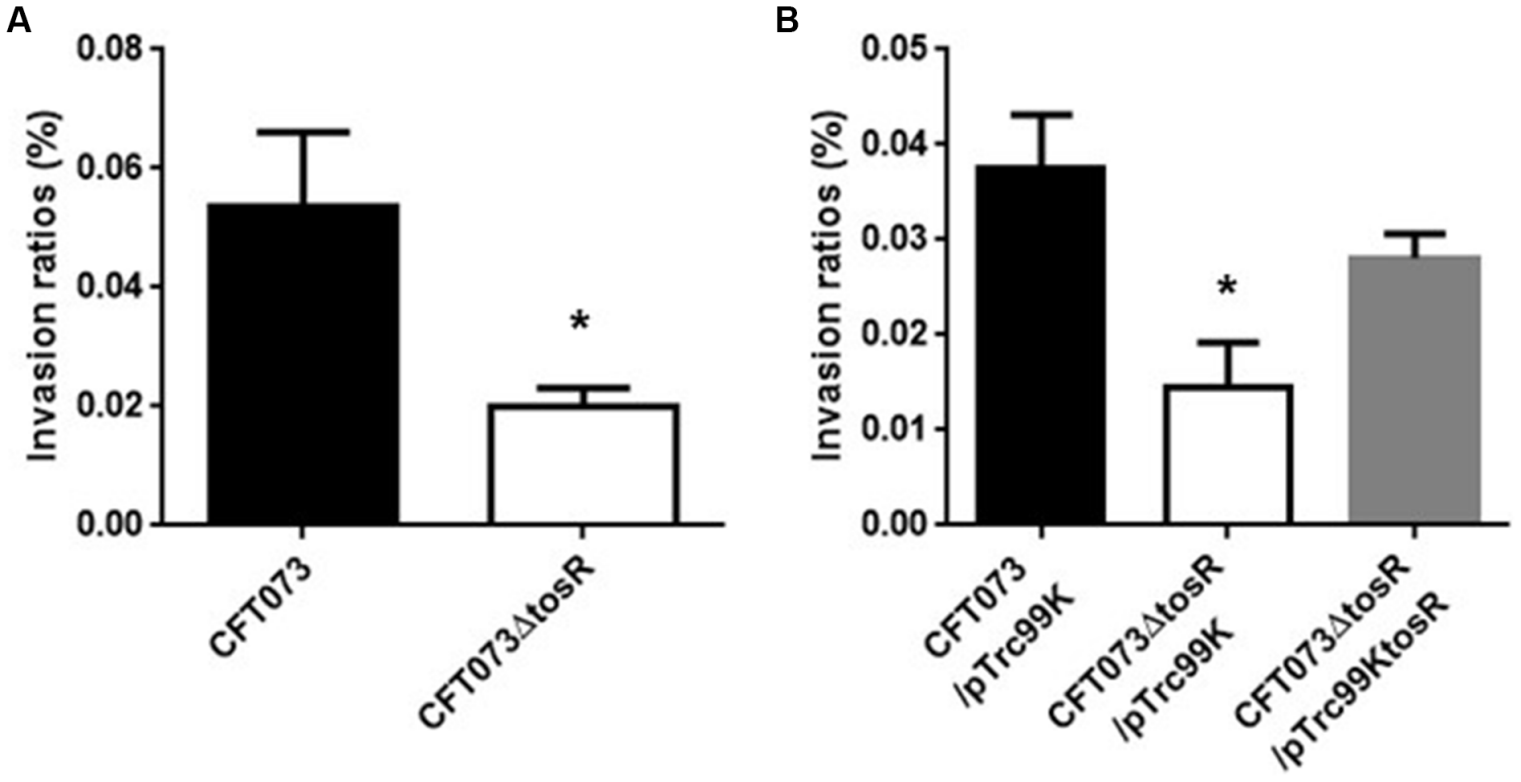
Internalization in bladder epithelial cells (HTB-9) of the parent strain (CFT073) and the *tosR* mutant **(A)** or the parent strain and the *tosR* mutant carrying pTrc99K or pTrc99KtosR **(B)**. Values are percent CFU of internalized bacteria relative to total bacterial cell numbers. Data are means from three independent experiments; error bars indicate standard deviations. *, *P*,0.05 relative to values for CFT073 **(A)** or CFT073/pTrc99K **(B)**.

**Figure 4:**
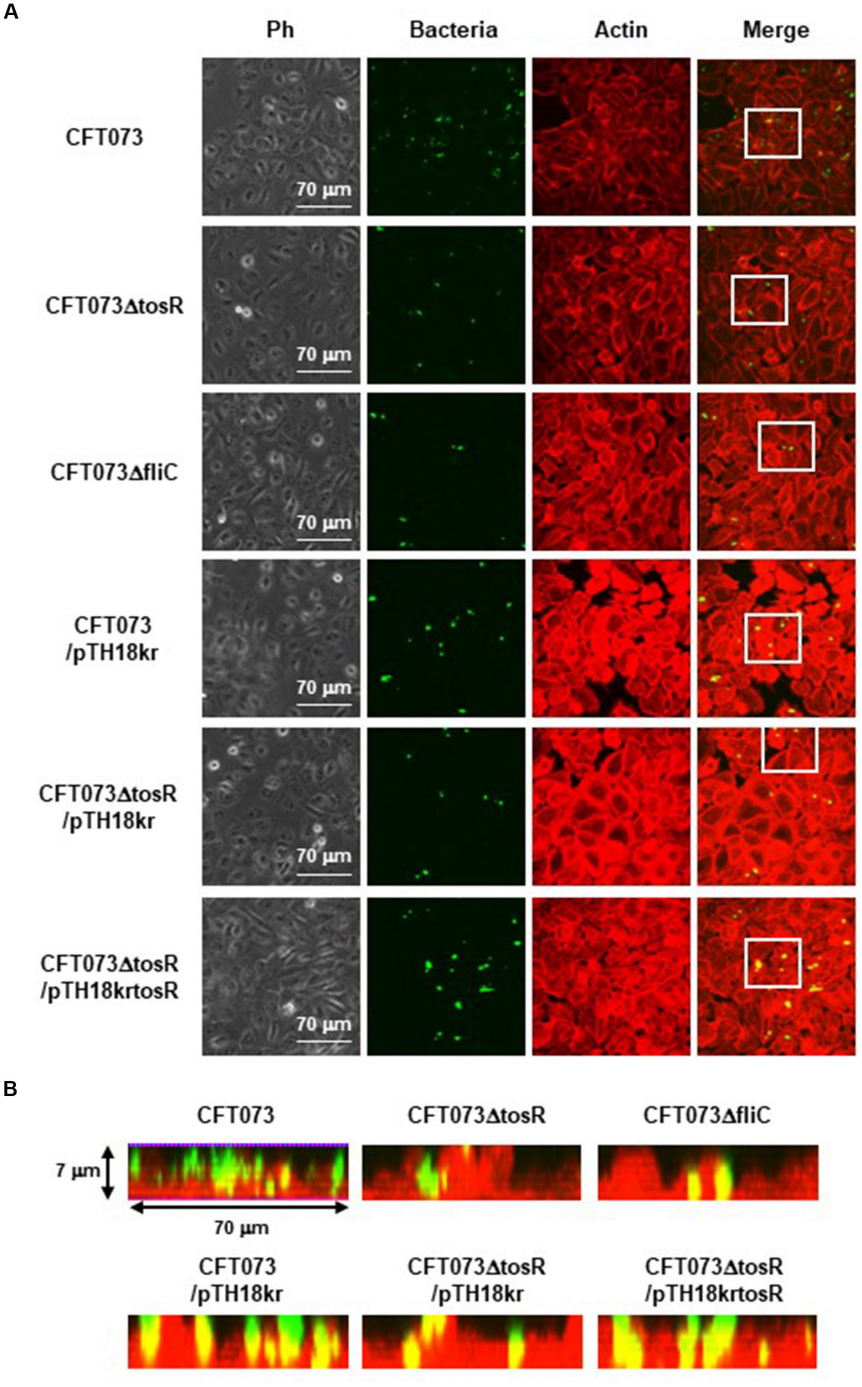
Aggregation within HTB-9 for the parent strain, the *tosR* mutant, the *fliC* mutant and the *tosR* complementation strain. Bacteria carrying a green fluorescence protein (GFP) expression plasmid, pTurboGFP-B, and HTB-9 cells (Actin) stained with rhodamine-phalloidin were imaged with green and red fluorescence, respectively, using a 60x objective. Images were taken from above, and cross-sectional images correspond to the white boxes. Ph: Phase contrast images. We performed this experiment twice, then similar results were obtained.

The biofilm forming ability of UPEC is closely related to invasion into and aggregation within urinary tract cells (Hirakawa et al., 2019). Therefore, we infected bladder epithelial cells with the parent strain and the *tosR* mutant, and compared the percentage of bacteria that entered the cells by gentamicin assay. The *tosR* mutant had a 2.7-fold lower value than the parent strain (0.020 +/− 0.002% vs. 0.054 +/− 0.009%, respectively; Figure 3A). Since there was no significant difference in total CFU/mL between the parent and mutant strains (CFU/mL of both strains were 1.2 × 10^8^), the reduction in invasion efficiency caused by the *tosR* deletion was not due to a growth defect. The complementation of the *tosR* mutant with pTrc99KtosR plasmid increased invasive ability (Figure 3B). We constructed a parent strain and a *tosR* mutant carrying a GFP-expressing plasmid. By inoculating each strain within bladder epithelial cells, we imaged UPEC cells by GFP fluorescence. In the parent strain, aggregated bacteria were observed in some places in the host cells (Figures 4A,B). On the other hand, the *tosR* mutant exhibited clearly fewer aggregates than the parent strain (Figures 4A,B). We have also confirmed that introduction of the *tosR* complementation plasmid pTH18krtosR into the *tosR* mutant restores the defective aggregation ability (Figures 4A,B). These results indicate that the *tosR* gene contributes to UPEC aggregation in urinary tract cells.

### The *tosR* mutant exhibits lower motility followed by a lower flagellar expression, than the wild-type parent

**Figure 5:**
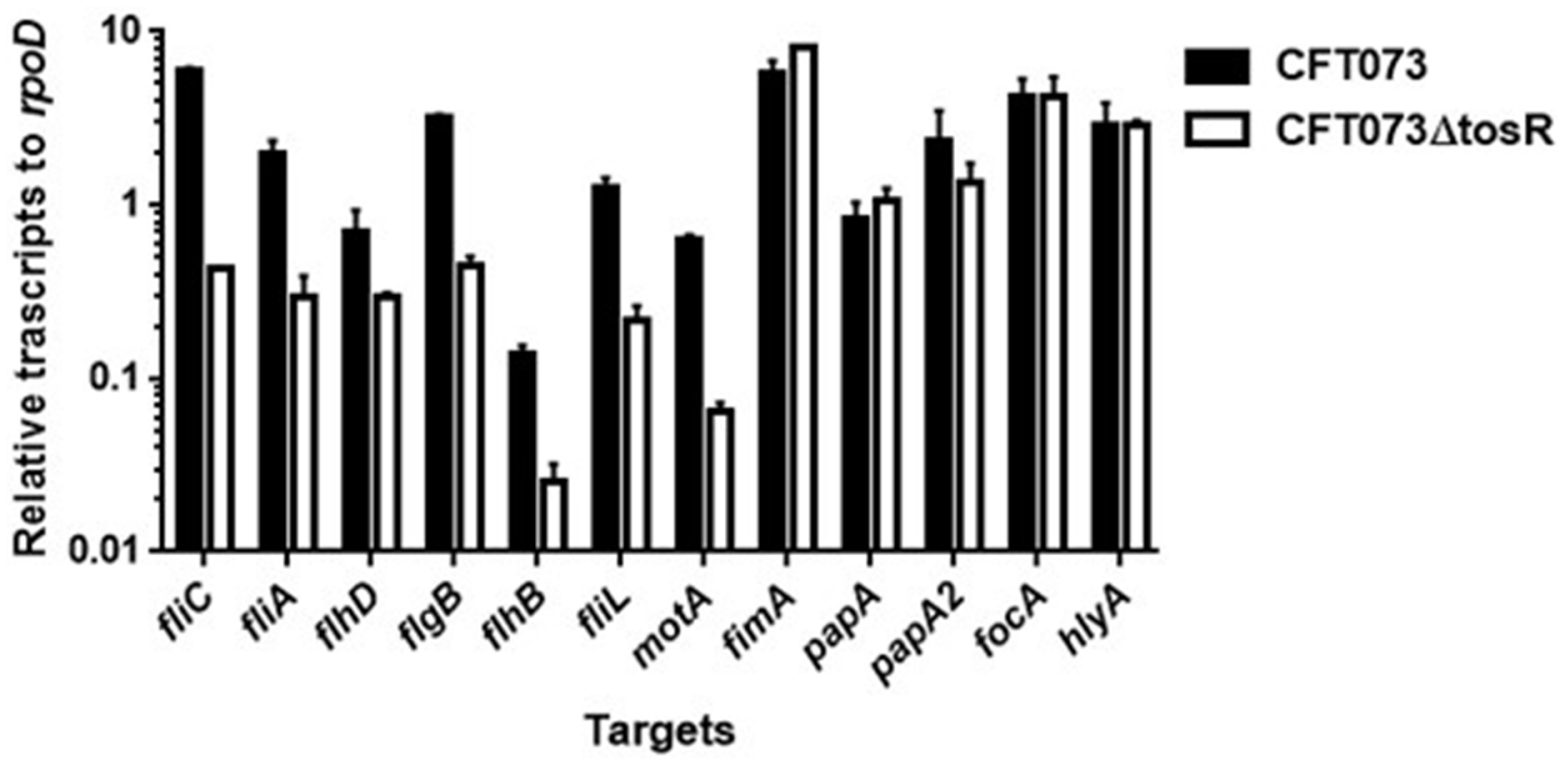
Transcript levels of flagellum-related and fimbrial genes and *hlyA* in the parent strain (CFT073) and the *tosR* mutant. Transcript levels were determined relative to that of *rpoD*. Data are means for two biological replicates; error bars indicate the ranges.

UPEC fimbriae are important for aggregation in uroepithelial cells. Since the *tosR* gene is presumed to encode a PapB/FocB family transcriptional regulator, we hypothesized that the reduced aggregation ability due to TosR defect may be due to reduced expression of fimbriae. Type 1 fimbriae agglutinate guinea pig erythrocytes in the absence of mannose, i.e., their agglutination activity is mannose-sensitive, and P fimbriae can agglutinate human erythrocytes regardless of the presence of mannose (Hagberg et al., 1981; Korhonen et al., 1985). We measured the activity of type 1 and P fimbriae in the presence and absence of mannose in the parent strain and the *tosR* mutant, using the agglutination titers of guinea pig and human erythrocytes as indicators. However, there was no difference in the titer values exhibited by the parent strain and the mutant strain under either condition (Table 4). We also compared the transcript levels of fimbriae genes between the parent and *tosR* mutant strains by quantitative PCR analysis. Transcript levels of *fimA* and *papA/papA2*, genes encoding proteins for the main component of type 1 and P fimbriae, were not significantly different between the parent strain and the *tosR* mutant (Figure 5). This result was consistent with the results of the agglutination test using guinea pig and human erythrocytes. Transcript levels for the *focA* gene, which encodes F1C fimbria, were not significantly different between the parent and *tosR* mutant (Figure 5).

**Figure 6:**
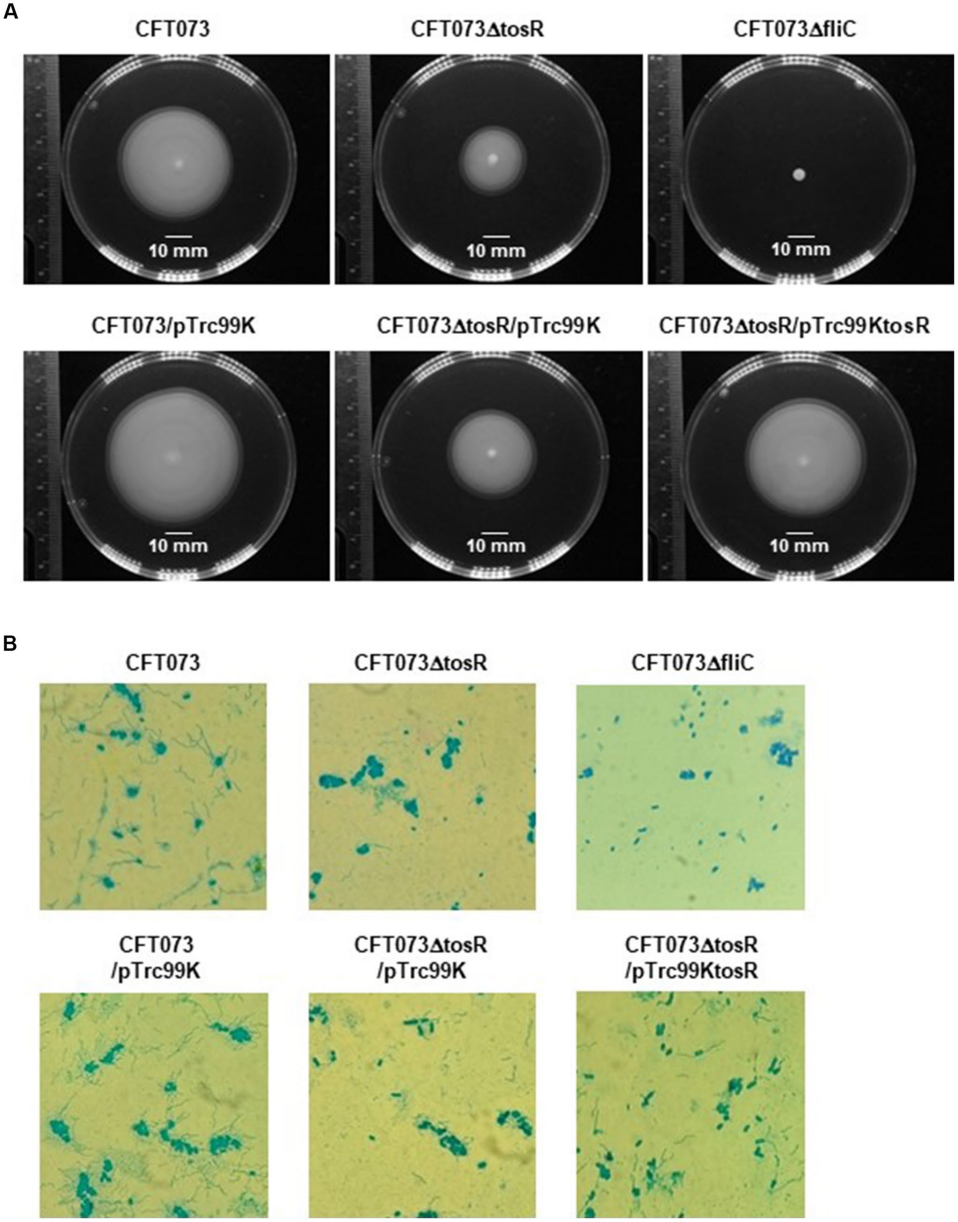
Motilities and flagellar production for the parent strain, the *tosR* mutant, the *fliC* mutant and the *tosR* complementation strain. **(A)** Bacterial migration on LB medium containing 0.3% agar. **(B)** Flagella and bacterial cells were stained with Victoria blue/tannic acid were pictured using a 100x objective. We performed this experiment twice, then similar results were obtained.

Flagella and flagellum-mediated motility of UPEC contribute to the aggregation of bacteria within urothelial cells (Hirakawa et al., 2019). Therefore, we tested whether the reduced aggregation ability caused by *tosR* deletion is due to reduced motility. Cultures of the parent strain and the *tosR* mutant were spotted on soft agar, and motility was compared by spreading of the bacteria, indicating that the *tosR* mutant was less motile than the parent strain (Figure 6A). As a control, the *fliC* mutant (CFT073∆fliC) showed no motility. Flagella are essential for motility. The results of flagellar staining showed that the *tosR* mutant had fewer flagella than the parent strain (Figure 6B). We also observed no flagellum in the *fliC* mutant. Furthermore, when the pTrc99KtosR plasmid was introduced into the *tosR* mutant, motility and flagellar production were increased (Figures 6A,B).

**Figure 7:**
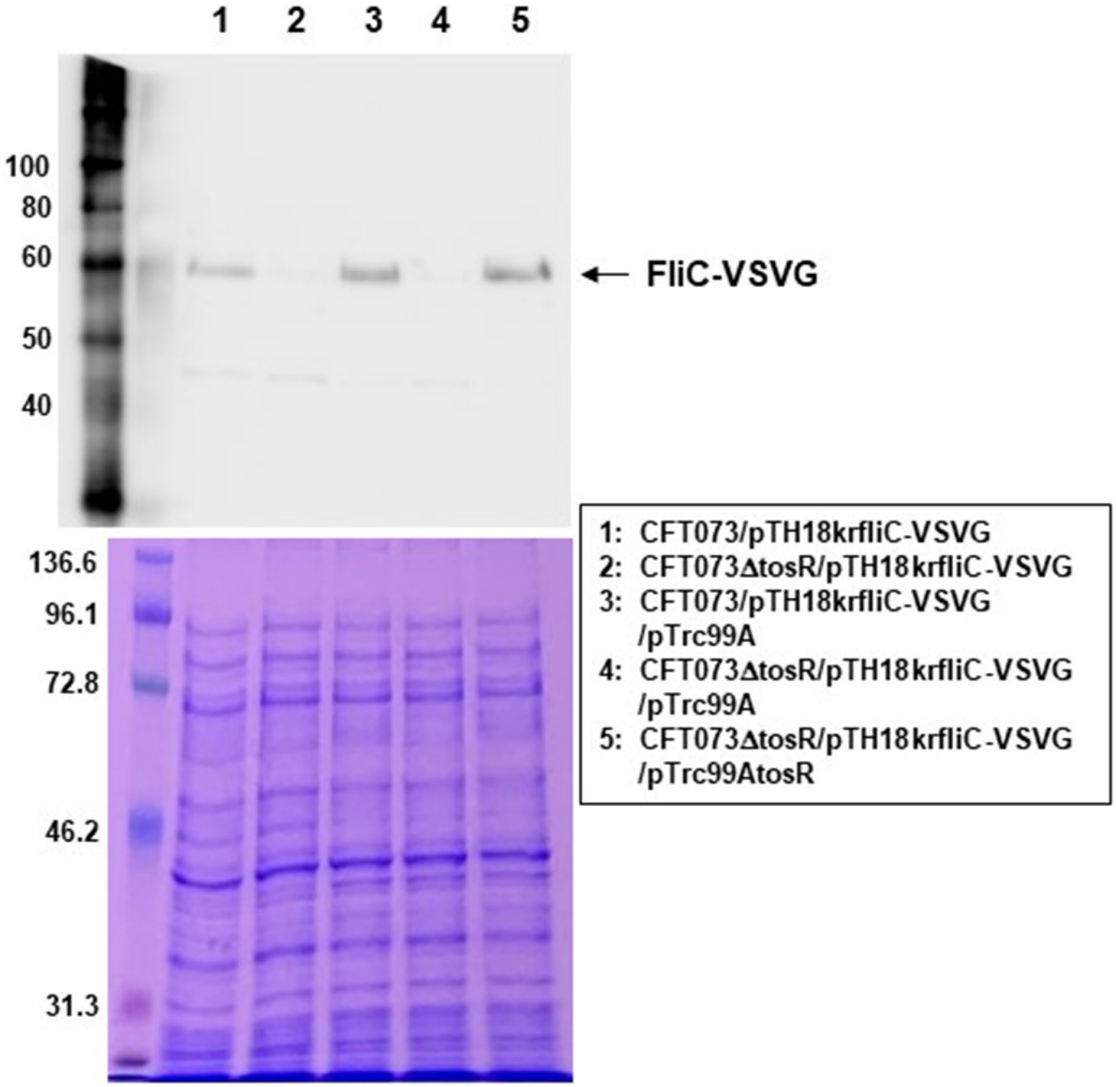
FliC expression in the parent strain (CFT073) and the *tosR* mutant. SDS-PAGE of cell lysates from the parent strain (CFT073) and the *tosR* mutant containing a VSVG-tagged FliC expression plasmid (pTH18krfliC-VSVG) and pTrc99A (empty vector) or pTrc99AtosR (*tosR* expression plasmid). Locations of molecular mass standards (in kilodaltons) are shown on the left. (Upper) VSVG-tagged FliC was visualized by probing with a VSVG antibody. (Lower) Proteins were stained with CBB. We performed this experiment twice, then similar results were obtained.

We examined the expression levels of flagellin FliC, a major constituent protein of flagella (Minamino and Imada, 2015), by Western blotting. We previously used a commercially available anti-flagellin antibody, but since no flagellin was detected even in the parent strain, we introduced the pTH18krfliC-VSVG plasmid into UPEC as an alternative method and expressed VSVG-tagged FliC as a recombinant protein (Hirakawa et al., 2021). Then, FliC was successfully detected using anti-VSVG antibody. The pTH18krfliC-VSVG plasmid contains the promoter and gene region of *fliC* inserted in the opposite direction of the *lac* promoter, which is upstream of the cloning site. In addition, the VSVG sequence is inserted just before the stop codon of *fliC*. Therefore, it is expected that strains carrying pTH18krfliC-VSVG will express C-terminally VSVG-tagged FliC, depending on the promoter of *fliC* itself. In this study, we used the same method with the pTH18krfliC-VSVG plasmid that we did previously. We compared the FliC-VSVG levels between CFT073 and CFT073∆tosR strains carrying the pTH18krfliC-VSVG plasmid. Western blotting results showed that the amount of band signal corresponding to FliC-VSVG expression from CFT073∆tosR was clearly lower than the CFT073 strain (Figure 7). In addition, when the *tosR* expression plasmid pTrc99AtosR was introduced into the CFT073∆tosR strain, the FliC-VSVG level was elevated. We note that the effect of *tosR* deletion on reduced FliC-VSVG levels appears more pronounced than the effect of reduced flagellar production and motility. This may be due to the low sensitivity of the VSVG antibody or to differences in culture conditions (For Western blotting, the bacteria were cultured in liquid, whereas for motility assays and flagellar staining, the bacteria were cultured on solid medium.).

We measured the transcript levels of *fliC* and its regulatory genes. Consistent with the Western blotting results, the transcript level of *fliC* was lower in the *tosR* mutant than in the parental strain (Figure 6). Transcription of *fliC* is activated by the sigma factor FliA, which is activated by FlhD, a master regulator complex of flagellar expression and *flhD* is transcribed together with *flhC* as an operon (Soutourina and Bertin, 2003). We found that, like *fliC*, the transcript levels of *flhD* and *fliA* were approximately 2-and 8-fold lower in the *tosR* mutant than in the parental strain, respectively. In addition to the *fliC*, *fliA*, and *flhD* genes, the transcript level of the *hlyA* gene encoding hemolysin was also measured. However, no significant difference in the transcript level was observed between the parent and *tosR* mutant (Figure 6). Altogether, these results indicate that the reduction in aggregation ability due to *tosR* deletion is due to reduced motility associated with reduced flagellar production.

### TosR is an activator of *flhD* gene expression

We investigated in more detail the role of TosR in regulating flagellar expression and its regulatory mechanisms. FlhDC also activates the transcription of genes that encode the flagellar components FlgB, FlhB, and FliL while the transcription of *motA,* encoding the motor protein for flagellar rotation, is induced by *fliA* (Macnab, 1992; Fitzgerald et al., 2014). The transcript levels of these genes in the *tosR* mutant were lower than those in the parental strain (Figure 6). These results indicate that reduced expression of *flhDC* and *fliA* due to *tosR* deletion leads to decreased transcript levels of *flgB*, *flhB*, *fliL*, and *motA*.

**Figure 8:**
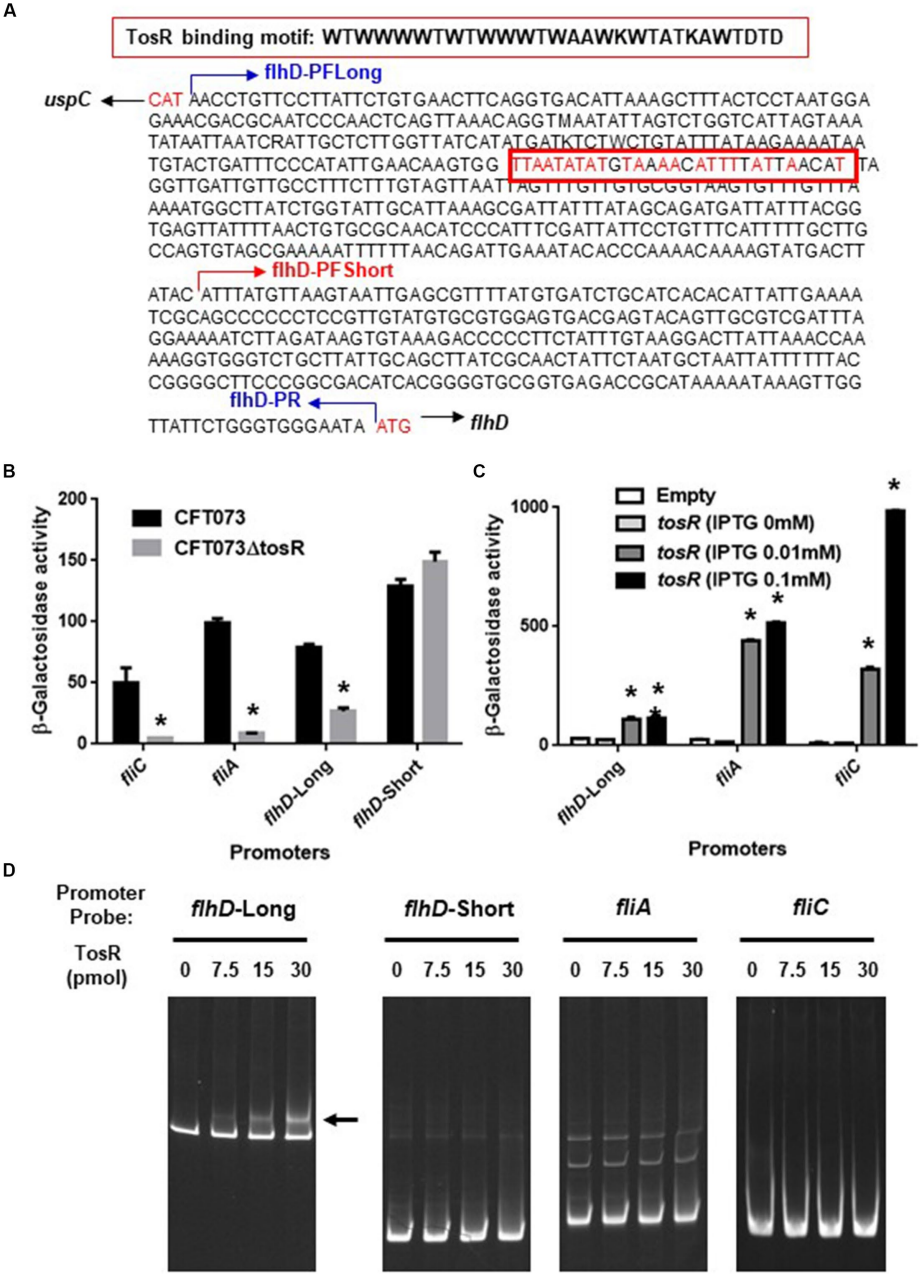
Promoter activities of *flhD*, *fliA*, and *fliC* and the TosR-binding to *flhD* upstream region. **(A)** DNA sequence upstream of *flhD*. flhD-PFLong, flhD-PFShort and flhD-PR are names of the primers and their locations. The red box is a DNA sequence to which TosR is presumed to bind. Nucleotides in red indicate nucleotides match within the TosR binding motif. **(B)** β-Galactosidase activities corresponding to *flhD*, *fliA*, and *fliC* promoter activities in the parent strain and the *tosR* mutant containing pNNflhD-Long, pNNflhD-Short, pNNfliA-P, or pNNfliC-P, the *lacZ* reporter plasmid. **(C)** β-Galactosidase activities corresponding to *flhD*, *fliA*, and *fliC* promoter activities in the *tosR* mutant carrying pTrc99K (empty) or pTrc99KtosR (IPTG-inducible *tosR* expression plasmid). Data are means from three independent experiments; error bars indicate standard deviations. *, *P*,0.01 relative to CFT073 or empty vector control. **(D)** Gel shift assay showing binding of TosR to the region upstream of *flhD*. The TosR protein (0, 7.5, 15 or 30 pmol) was added to reaction mixtures containing 0.3 pmol of DNA probe. We performed this experiment twice, then similar results were obtained. Promoters and DNA probes designated flhD-Long and flhD-Short contain the region from 769-bp (including the AT-rich site) and 300-bp (lacking the AT-rich site) upstream from the *flhD* start codon, respectively.

We also compared the promoter activities of *flhD*, *fliA*, and *fliC* in the parent and mutant strains. We determined the activity of each promoter by measuring the β-galactosidase activity corresponding to LacZ expression from the reporter plasmids pNNflhD-P-Long, pNNflhD-P-Short, pNNfliA-P, and pNNfliC-P. To evaluate *flhD* promoter activity, the pNNflhD-P-Long and pNNflhD-P-Short plasmids were used. TosR has been reported to bind to an AT-rich DNA sequence consisting of “WTWWTWTWWTWAAWKWTATKAWTDTDTD” (Luterbach et al., 2018; Figure 8A). We found a sequence similar to the TosR binding motif upstream of the *flhD* start codon at positions 550–578. The pNNflhD-P-Long plasmid is a reporter construct consisting of a 769-bp DNA fragment containing the entire intergenic region between *flhD* and its upstream gene, *uspC*. The pNNflhD-P-Short plasmid includes a region only 300 bases upstream of *flhD* lacking the AT-rich region, with a promoterless *lacZ* reporter gene. The promoter activity measurements revealed that these promoter activities from pNNflhD-P-Long, pNNfliA-P, and pNNfliC-P, but not pNNflhD-P-Short, were significantly lower in the *tosR* mutant than in the parent strain (Figure 8B). We also introduced the IPTG-inducible *tosR* expression plasmid pTrc99KtosR with each reporter plasmid, and measured LacZ activities. A concentration-dependent increase in LacZ activities was observed with IPTG (Figure 8C). We note that a growth defect was observed in the IPTG 0.1 mM culture similar to the biofilm assay, however a high degree of increased promoter activity was observed. These results suggest that the expression of *tosR* promotes the promoter activity of *flhD*, *fliA*, and *fliC*.

We examined the DNA binding ability of TosR by gel shift assay. In the presence of 7.5 pmol of TosR-His_8_ protein, a band shift was observed in the DNA probe containing the AT-rich region upstream of *flhD* (Figure 8D). We used a DNA probe containing only a region 300 bases upstream of *flhD* that lacked the AT-rich region. We also examined the binding ability of TosR to DNA probes containing the upstream 300 bases of *fliA* and *fliC*. As a result, no band shift in these DNA probes was observed even when 30 pomol of TosR-His_8_ was present (Figure 8D). These results indicate that TosR is an activator that binds to the AT-rich region upstream of *flhD* and stimulates the promoter activity of *flhD*.

## Discussion

**Figure 9:**
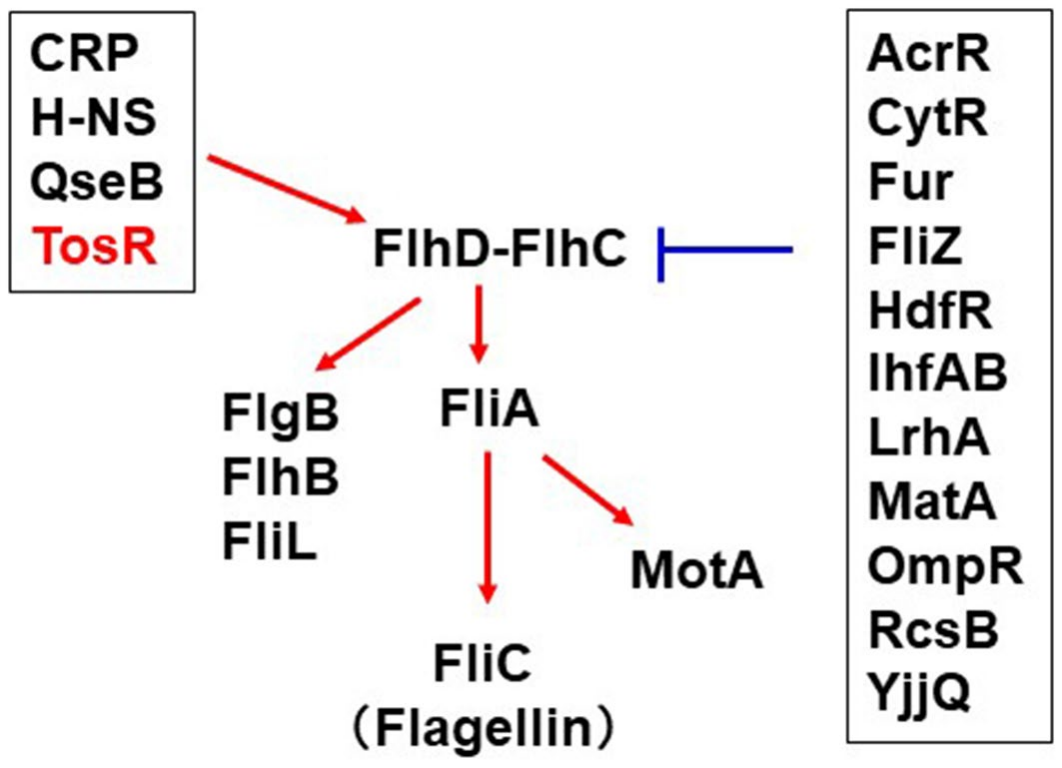
Overview of the regulatory mechanism of *flhD* expression and contribution of this study. The red arrows positively regulate *flhDC* transcription while the blue bars indicate repression of *flhDC* transcription. The information on the regulator groups framed in black was taken from RegulonDB (Tierrafria et al., 2022). CytR recently characterized in our study was added (Hirakawa et al., 2020). TosR was clarified in this study.

Flagella of UPEC contribute to bacterial aggregation leading to IBC formation in host epithelial cells (Hirakawa et al., 2019, 2021). On the other hand, flagellin binds to Toll-like receptor-5 and activate the host immune system (Miao et al., 2007). Therefore, the expression level of UPEC flagella must be tightly regulated within the infected host. Expression of the *fliC* gene, which encodes the major flagellar component flagellin, is activated by the sigma factor FliA (Soutourina and Bertin, 2003). Expression of *fliA* is activated by the master regulator of flagellar expression, FlhD-FlhC. Expression of *fliC* is also controlled by a number of regulatory proteins including CRP, LrhA, H-NS, QseB, RcsB, OmpR, Fur, TosEF and CytR (Bertin et al., 1994; Shin and Park, 1995; Soutourina et al., 1999; Lehnen et al., 2002; Francez-Charlot et al., 2003; Clarke and Sperandio, 2005; Engstrom et al., 2014; Kurabayashi et al., 2016; Hirakawa et al., 2020). In addition, the database RegulonDB revealed a set of potential regulators for FlhDC expression (Tierrafria et al., 2022; Figure 9). This study showed that TosR is also involved in the regulation of flagellar expression.

TosR has been characterized as a PapB/FocB family transcriptional regulator that controls the expression of the nonfimbrial adhesin TosA (Engstrom and Mobley, 2016). TosR has also been shown to bind to AT-rich DNA sequences (Luterbach et al., 2018). Previously, a transcriptome analysis identified a set of genes whose transcript levels increase or decrease upon TosR overexpression (Luterbach et al., 2018). Interestingly, no flagellar gene was identified in that study. We noticed the presence of an AT-rich sequence upstream of the *flhDC* operon that resembles the TosR binding motif. Our gel shift assay revealed that TosR bound to a DNA probe containing the AT-rich sequence upstream of its *flhD*, while it did not bind to the DNA probe lacking the AT-rich sequence. Furthermore, in promoter assays, the LacZ reporter construct of the *flhD* promoter containing the AT-rich sequence showed reduced LacZ activity upon *tosR* deletion and increased LacZ activity upon *tosR* overexpression. In contrast, no effect of *tosR* deletion was observed for the LacZ reporter construct lacking the AT-rich sequence. These results suggested that TosR positively regulates *flhDC* expression.

Deletion of *tosR* causes reduced flagellar production. This is supported by the finding that *tosR* deletion reduces motility. However, a study conducted by Engstrom et al., showed that the *tosR* mutant exhibited motility comparable to that of the parental strain (Engstrom et al., 2014). Bacterial motility can be affected by even small differences in medium, temperature, and agar concentration. In Engstrom’s assay, the bacteria were stabbed on agar medium without yeast extract and evaluated after 17 h, whereas in our assay, the bacterial cultures were spotted on agar medium containing yeast extract and evaluated after 9 h. Our assay seems to inoculate more bacteria than Engstrom’s assay. If we incubate for 17 h as in the Engstrom’s assay, both the parent and mutant strains will migrate to the edge of the plate, making it impossible to compare motility between the two strains. We believe that the difference from the Engstrom’s assay is due to the difference in medium and inoculation method.

The *tosR* gene, together with the *tosA* gene, is encoded on a pathogenicity island on the chromosome of CFT073, and this pathogenicity island containing the *tosR* and *tosA* genes is also found in many UPECs besides CFT073 (Engstrom et al., 2014). Therefore, TosR may play a significant role in the regulation of flagellar expression in UPEC.

TosR is known to repress the expression of P fimbria while promoting the expression of Auf fimbria and Curli, amyloid-like fibers (Luterbach et al., 2018). Furthermore, TosR regulates *tosA* expression in a TosR level-dependent manner (Engstrom and Mobley, 2016). P and Auf fimbriae, Curli and TosA are involved in adhesion to urinary epithelial cells. The *tosR* mutant used in this study is actually less virulent to uroepithelial cells than the parent strain, although it is presumed to have increased P fimbria and TosA levels while expressing less Auf fimbria and Curli, at least compared to the parent strain. Colonization of UPEC within urinary tract epithelial cells is linked to UPEC’s ability to form biofilm (Hirakawa et al., 2019). UPEC colonization and biofilm formation in uroepithelial cells are positively correlated with the flagellar expression (Hirakawa et al., 2019, 2021). Taken together, we speculate that the reduced expression of Auf fmbria and Curli in the *tosR* defective strain, as well as the effect of reduced UPEC colonization and biofilm formation capacity due to reduced flagellar expression, mask the effect of increased P fimbria and TosA levels.

The region upstream of *flhD* where TosR acts is more than 500 bases away from the *flhD* start codon, and it is still unclear how TosR promotes *flhD* transcription by binding to a region far from the *flhD* promoter. Nucleoid-associated proteins, such as H-NS, are known to bind to DNA and modulate the local nucleoid structure, thereby regulating the expression of genes far away (Dorman, 2004). TosR is thought to regulate gene expression in a similar manner to nucleoid-associated proteins (Engstrom and Mobley, 2016). Therefore, it is possible that TosR binding to *flhD* promotes transcription through modulation of the local nucleoid structure.

Previous studies showed that *tosR* expression is repressed by H-NS *in vitro* (under 37°C, LB medium conditions), and its repression is alleviated in urinary tract infection mice (Vigil et al., 2011, 2012; Engstrom et al., 2014). However, in this study, the effect of *tosR* deletion on flagellar expression was observed at 37°C in LB medium, implying that *tosR* is expressed *in vitro* to some extents. The regulatory mechanism of *tosR* expression needs to be elucidated to understand the mechanism of virulence induction in UPEC, including flagellar expression.

In addition to TosR, a number of PapB/FocB family transcriptional regulators, including SfaB, DaaA, FaeB, FanA, FanB, ClpB, PefB, and AfaA, have been characterized in *E. coli* and *Salmonell*a (Xia and Uhlin, 1999; Holden et al., 2001). While they have been described to regulate the expression of various fimbriae and other adhesin-related genes (Spurbeck et al., 2011), they have not been shown to regulate the flagellum. We provide evidence that TosR is a positive regulator of flagellar expression. We believe that our findings suggest additional possible functions for the PapB/FocB family of transcriptional regulators and provide further insight into the mechanisms of flagellar expression and pathogenesis in UPEC.

## Data availability statement

The original contributions presented in the study are included in the article/Supplementary material, further inquiries can be directed to the corresponding author.

## Author contributions

HH, HTA, IK, and HTO designed the research and wrote the manuscript. HH, MS, KN, MT, HM, AT, and KS performed the experiments. HH, IK, and HTO analyzed the data.

## Funding

This research was funded by the Japan Society for the Promotion of Science (JSPS) “Grant-in-Aid for Scientific Research (B)” (Grant No. 22H02864 to HH and KS), Japan Agency Research and development [AMED] (Grant No. 22fk0108604h0902 and 22wm0225008h0203 to HTO), and Health Labour Sciences Research Grant (Grant No. 21KA1004 to HTO).

## Conflict of interest

The authors declare that the research was conducted in the absence of any commercial or financial relationships that could be construed as a potential conflict of interest.

## Publisher’s note

All claims expressed in this article are solely those of the authors and do not necessarily represent those of their affiliated organizations, or those of the publisher, the editors and the reviewers. Any product that may be evaluated in this article, or claim that may be made by its manufacturer, is not guaranteed or endorsed by the publisher.
